# Contraceptive and condom use among women from key populations in Myanmar

**DOI:** 10.1186/s12954-026-01520-z

**Published:** 2026-09-12

**Authors:** Maxim Kan, Carl Fredrik Sjöland, Khine Wut Yee Kyaw, Ruby Killingsworth, Sai Phyo Zarni, Martin Kåberg, Aung Yu Naing, Anna Mia Ekström

**Affiliations:** 1 Department of Global Public Health, Karolinska Institutet, Solna, Sweden; 2 Public Health Agency of Sweden, Solna, Sweden; 3 National Drug and Alcohol Research Centre (NDARC), University of New South Wales, Sydney, Australia; 4 The Stockholm Needle and Syringe Programs, Stockholm Centre for Dependency Disorders, Stockholm, Sweden; 5 Asian Harm Reduction Network and Best Shelter, Yangon, Myanmar; 6 Department of Infectious Diseases/Venhälsan, Södersjukhuset, Stockholm, Sweden; 7 Department of Clinical Science and Education, Södersjukhuset, Karolinska Institutet, Stockholm, Sweden

**Keywords:** Injecting drug use, HIV, Women who sell sex, Women who use and inject drugs, Contraceptive use, Condom use, Marginalized women, SRHR, Myanmar

## Abstract

**Background:**

Contraceptive and condom use remain low among women in marginalized communities, including those who sell sex, use or inject drugs, or are sexual partners of people who use or inject drugs (key populations). This study examines how selling sex and drug use relate to contraceptive and condom use with different partner types in Myanmar, alongside structural risk contexts.

**Methods:**

We analyzed a cross-sectional survey among women from key populations (women who sell sex, use drugs, or are sexual partners of men who use drugs) recruited through community-based organizations across four sites in Northern and Eastern Myanmar. The analysis focused on women of reproductive age (15–49 years). Participants completed standardized questionnaires assessing contraceptive use in the past 12 months and condom use with partner types in the past 3 months. Descriptive and bivariate analyses were conducted, and multivariable Poisson regression models estimated adjusted prevalence ratios (aPRs) for contraceptive and condom use in relation to selling sex, drug use, and selected covariates.

**Results:**

In multivariable analyses among 336 women, those reporting drug use only (aPR 0.68, 95% CI 0.48–0.95) had lower contraceptive uptake compared with women with no personal experience of selling sex or drug use. Women who had never used drugs were more likely to use contraception than opioid users (aPR 0.64, 95% CI 0.43–0.95) or stimulant users (aPR 0.72, 95% CI 0.57–0.92). Work-provided housing was associated with higher contraceptive use (aPR 1.43, 95% CI 1.01–2.05). This association was strongest among individuals selling sex, for whom such housing may represent brothel environments that facilitate access to contraceptives. Experience of selling sex was associated with higher condom use with casual partners, whereas drug use only or neither experience was associated with lower condom use with casual partners, highlighting prevention gaps outside sex-work-focused outreach.

**Conclusion:**

Among women from key populations in Myanmar, contraceptive and condom use were determined by structural factors and risk contexts. Drug use was associated with lower contraceptive uptake, while experience of selling sex and work-provided housing were linked to higher use, likely reflecting differential access to outreach-based services. Scaling up reproductive health and HIV-prevention services within harm reduction and outreach, while addressing housing insecurity, is essential to improve outcomes in these populations.

## Introduction

Access to contraception is a cornerstone of sexual and reproductive health and rights (SRHR), yet disparities in uptake remain pronounced among marginalized women. Women who sell sex, use drugs, or are sexual partners of people who use drugs face compounded barriers to optimal reproductive health, including stigma, criminalization, and limited access to care [1–5]. Women who belong to such marginalized groups are often excluded from national family planning programs and surveys, despite their elevated risks of unintended pregnancy and sexually transmitted infections (STIs) including HIV [2, 3, 5].

Research on harm reduction and SRHR increasingly emphasizes that reproductive health behaviors among marginalized women are shaped by broader “risk environments,” including criminalization, housing insecurity, stigma, mobility, policing practices, and uneven access to integrated health services [6–9]. Within these environments, engagement with outreach programs and peer-led interventions may facilitate access to condoms and contraceptives, while social exclusion and unstable living conditions may reduce continuity of care and reproductive autonomy [10–13].

International evidence suggests that women who use drugs experience lower contraceptive uptake and higher unmet reproductive health needs compared with the general population, driven by stigma, criminalization, unstable housing, and fragmented health systems [14–17]. Studies among women who sell sex have shown that peer-led outreach and targeted HIV prevention programs may improve condom use and access to SRHR services, although coverage remains uneven, particularly in conflict-affected and criminalized settings [10, 13, 18–22]. However, most existing evidence originates from high-income or more stable settings, with limited data from conflict-affected regions in Southeast Asia.

These challenges are particularly important in Myanmar’s border and conflict-affected regions, where ongoing instability, displacement, and reliance on NGO-led harm reduction programs contribute to uneven access to contraception, HIV prevention, and broader SRHR services. In Myanmar, HIV prevalence in the general population is estimated at approximately 0.9% [23], yet this burden is substantially higher among marginalized groups, reaching 4.9% among women who sell sex and more than 20% among people who inject drugs [24], underscoring profound inequities in sexual and reproductive health risks. The national prevalence for use of modern contraceptive methods (including oral contraception, injectable contraceptives, implants, vaginal rings and intrauterine devices) among married women was approximately 51% in 2015–2016, but use remains unevenly distributed [25–30]. Previous studies have identified age, parity, education, economic status, and geography as key determinants of contraceptive use among married women in Myanmar, with substantial urban–rural and regional variation [28–30]. However, national surveys primarily focus on married women and do not capture information on more marginalized women at high risk of unwanted pregnancies and STIs, particularly those who sell sex and/or use drugs, groups that constitute a substantial proportion of women of reproductive age in some regions such as the Golden Triangle, though there are no reliable estimates. As a result, current national evidence provides limited guidance for reproductive health policies targeting women outside conventional household-based surveillance systems.

By examining both contraceptive use and condom use across different partner types, this study explores how risk-group membership, drug use patterns, and structural conditions shape reproductive and HIV-prevention behaviors among women underserved by national surveillance systems.

## Data and methods

### Study design and setting

This cross-sectional study draws on survey data collected in November–December 2022 among women who sell sex, use drugs, or are sexual partners of people who use drugs in selected sites within Kachin and Shan State, Myanmar. The study sites were purposively selected to represent settings with a high prevalence of drug use, selling sex, and related SRHR vulnerabilities, and were chosen based on their accessibility for community-based outreach and their relevance to populations disproportionately affected by gender and structural inequities in reproductive health. The four recruitment sites were located in conflict-affected areas of Northern and Eastern Myanmar and were selected to capture variation in program implementation contexts and population mobility. Sites differed in terms of levels of ongoing instability, accessibility of health services, and intensity of outreach activities delivered through implementing partners. Due to confidentiality considerations, specific site locations are not reported; however, sites were broadly categorized by state (Kachin and Shan) to preserve anonymity while allowing regional comparison. Individual locations are anonymized and are referred to as Site 1 (Kachin State), Site 2 (Kachin State), Site 3 (Kachin State), and Site 4 (Shan State) in subsequent tables and analyses.

### Study population and sampling

Participants were recruited through community-based organizations (Asian Harm Reduction Network–Best Shelter (AHRN-BS)) that provide integrated harm reduction services, including women-focused interventions such as women-specific drop-in centers or corners and SRHR services, to a broad range of target populations. Participants were reached through peer-outreach workers using venue-based and snowball sampling strategies in line with standard outreach practice by the organization. The questionnaire was collaboratively developed by AHRN-BS staff together with researchers and clinicians at Karolinska Institute and aimed to take between 45 and 60 min to complete. It was refined through a series of pilot rounds prior to administration to participants. Eligible participants were individuals aged 15 years or older who self-reported current or previous experience of selling sex or use of non-injected or injected drugs, or who reported being a current sexual partner of a person who uses non-injected or injected drugs. Eligibility criteria were assessed through standardized interviewer-administered questionnaires at recruitment. No predefined recruitment quotas were applied by participant category or study site; recruitment reflected routine outreach practices and participant availability within each location. Multiple recruitment sites were included to capture variation in conflict setting, mobility, drug-use patterns, and access to harm reduction and SRHR services across Northern and Eastern Myanmar, and to improve representation of women from different marginalized populations and service contexts.

In Myanmar, both illicit drug use and activities related to selling sex are criminalized, contributing to stigma and barriers to healthcare access among affected populations. Informed consent was obtained from all participants prior to data collection. The study was approved by the Swedish Ethical Review Authority (dnr 2022-01321-01), and approval for Myanmar data handling and analysis by special permission from Karolinska Institute’s Resource Group for Ethical Review (dnr 2022-12-14). Ethical approvals permitted inclusion of participants under 18 years of age, using parental/guardian consent or self-consent for emancipated minors as appropriate.

A total of 429 respondents aged 15–76 years were recruited. The analytical sample was restricted to women of reproductive age (15–49 years). Of the 429 women surveyed, 361 met the age eligibility criteria and were included in descriptive and bivariate analyses. Multivariable analyses were conducted among 336 participants with complete data on the outcome variables and core covariates. Sample size varied across the condom use models because condom use was assessed only among participants who reported having the specific partner type under analysis (main, regular, casual known, or casual unknown).

### Data collection

Data were collected through an interviewer-administered structured questionnaire in Burmese, covering sociodemographic characteristics, reproductive health behaviors, and experiences related to drug use and/or selling sex. Interviews were conducted in private, unobserved, and secure settings, including drop-in centers, outreach service sites, clinics, participants’ homes, or private residential areas within sex selling venues. Interview locations were determined by participants as safe and without risk of being overheard, with alternative locations offered if any discomfort or hesitation was expressed, to ensure confidentiality, comfort, and the reliability of responses. Interviews were conducted by trained peer outreach workers and field staff affiliated with AHRN–BS, who were actively engaged in community-based harm reduction and sexual and reproductive health service delivery. As a result, many interviewers had prior contact with participants through outreach activities, which may have facilitated rapport and disclosure. The research team based at Karolinska Institutet provided technical oversight, training, and supervision but did not directly conduct interviews.

### Variables

#### Outcome variables

The primary outcome was contraceptive use in the past 12 months, defined as self-reported use of any modern contraceptive method (oral contraceptive pill, injectable, implant, intrauterine device, condom, or sterilization) for pregnancy prevention. This was coded as a binary variable (1 – used any method in the past 12 months; 0 – no use). Condom use was assessed as a secondary outcome and measured separately for different types of sexual partners. Participants were asked whether they had used a condom during sexual intercourse in the past three months with each of the following partner types: main partner, regular partner, casual known partner, and casual unknown partner. For each partner type, condom use was coded as a binary variable (1 = used; 0 = not used). Analyses were restricted to participants who reported having the corresponding partner type during the reference period.

#### Explanatory variables

Sociodemographic and structural covariates included *education* (categorized as no formal education, primary school (elementary level), secondary school (high school level), tertiary school, and university level), *housing security* (own housing (self-owned, family-owned, or privately rented accommodations); work-provided housing (for the majority of participants in this category (96% of whom were engaged in selling sex), this primarily indicated residence in a brothel or other sex work venue. For the small proportion of participants not engaged in sex work, this may have represented other forms of employer-provided accommodation); other/less secure housing (included residence in internally displaced person camps, temporary arrangements (e.g., sleeping on someone’s sofa or floor), squatting, having no fixed address (e.g., street, park), or staying in a drug rehabilitation center), *religion* (Christianity vs. Buddhism), *ethnicity* (Kachin, Bamar and Other incl. Shan and Pa’O)), *marital status* (currently married vs. not), *age group* (15–24, 25–34, 35+), and *survey/clinic site* (Site 1 (Kachin State), Site 2 (Kachin State), Site 3 (Kachin State), Site 4 (Shan State) – for confidentiality and security reasons in conflict-affected areas, study sites are anonymized and reported by state rather than by specific location.). *Drug use and selling sex status* was categorized into four groups: neither, selling sex only, drug use only, and both selling sex and drug use. Drug use was categorized into four mutually exclusive groups based on self-reported current or past use of psychoactive substances: never used drugs, opioid use, stimulant use (primarily methamphetamine/amphetamine-type stimulants), and other drug use. The “other drug use” category included less commonly reported substances that could not be meaningfully analyzed separately due to small sample sizes. Information on both injected and non-injected drug use was collected; however, route of administration was not included in the present analyses because of limited statistical power for stratified analyses. An additional variable captured whether respondents were the *sexual partner* of a person who uses or injects drugs.

Because selling sex and drug use status was a nominal multi-category variable, bivariate associations were assessed using cross-tabulations rather than correlation coefficients. Selling sex and drug use status and the sexual partner variable were included simultaneously in multivariable Poisson regression models. Multicollinearity among all covariates, including selling sex and drug use status and sexual partnering, was assessed using variance inflation factors obtained from an equivalent linear regression model with the same set of predictors. All variance inflation factors were below 4 (mean = 2.21), indicating no evidence of problematic multicollinearity.

### Statistical analysis

We first conducted descriptive analyses of sample characteristics by contraceptive use status, using Pearson’s chi-squared tests for categorical variables. We then fitted multivariable Poisson regression models with robust standard errors to estimate prevalence ratios (PRs) for contraceptive use and condom use. All models controlled for education, housing, ethnicity, experience of drug use/selling sex status or drug group, age group, site, and being sexual partner of a person who uses drugs. Reference categories for *drug use and selling sex status variable* were selected based on the conceptual relevance of each outcome. For contraceptive use, women with neither selling sex nor drug use experience were used as the reference group to represent a baseline with lower exposure to structural barriers affecting pregnancy prevention. For condom use, women engaged in selling sex only were used as the reference group, reflecting a population with comparatively greater exposure to HIV/STI prevention services and condom promotion. This outcome-specific approach improves interpretability and aligns comparisons with the distinct prevention contexts of pregnancy and HIV/STI risk. Statistical significance was set at *p* < 0.05. Analyses were conducted in Stata MP 19.5 (StataCorp, College Station, TX).

## Results

A total of 361 women of reproductive age (15–49 years) were included in descriptive and bivariate analyses. Participants were recruited across four study sites, including three sites in Kachin State (Sites 1–3) and one site in Shan State (Site 4). Recruitment numbers by site were as follows: Site 1 (*n* = 68), Site 2 (*n* = 179), Site 3 (*n* = 61), and Site 4 (*n* = 53). Site-level differences should be interpreted cautiously, as study sites likely reflect variation not only in geography, but also in local conflict conditions, mobility patterns, outreach intensity, service availability, and composition of recruited populations.

### Bivariate analysis

Table 1 presents bivariate associations between contraceptive use and participant characteristics. Compared with women who used contraception, women who did not use contraception were more likely to be unemployed (40.1% vs. 29.0%, *p* = 0.027), have less secure housing (46.9% vs. 33.2%, *p* < 0.001), and belong to the oldest age group (35 + years: 51.7% vs. 22.9%, *p* < 0.001).

Not using contraception was also more common among women whose sexual partner injected drugs (59.1% vs. 45.7%, *p* = 0.016). Significant differences were observed by experience of selling sex or drug use (*p* < 0.001): among women who used contraception, selling sex only was more frequent (43.5% vs. 21.1% among non-users), whereas among women who did not use contraception, drug use only (33.3% vs. 15.0%) and combined selling sex and drug use (22.4% vs. 17.8%) were more common.

Similarly, women who had never used drugs were more likely to use contraception (67.3%) than not (44.2%), while opioid use was more common among women who did not use contraception than among those who did (23.8% vs. 8.9%, *p* < 0.001). Contraceptive use did not differ significantly by religion, marital status, or ethnicity. However, contraceptive use varied across study sites (*p* = 0.025), with a higher proportion of women using contraception observed in Site 2 (Kachin State) and Site 4 (Shan State). These differences likely reflect variation in service availability, intensity of outreach activities, and proximity to NGO-supported sexual and reproductive health and harm reduction services across sites, as well as differences in population mobility and conflict intensity between states.

**Table 1:** Bivariate associations between contraceptive use and participant characteristics, Myanmar (*N* = 361)

Factor		Contraceptive use		
	Level	No	Yes	*p*-value
N		147	214	
Religion	Christianity	44 (30.0%)	48 (22.4%)	0.089
	Buddhism	100(68.0%)	165 (77.1%)	
	Missing	3 (2.0%)	1(0.5%)	
Employment	No	59 (40.1%)	62 (29.0%)	0.027
	Yes	88 (59.9%)	152 (71.0%)	
Education	No formal education	13 (8.8%)	17 (7.9%)	0.037
	Primary School	52 (35.4%)	49 (22.9%)	
	Secondary School	43 (29.3%)	67 (31.3%)	
	Tertiary School	36 (24.5%)	67 (31.3%)	
	University level	3 (2.0%)	14 (6.5%)	
Marital status	Not currently married	63 (42.9%)	101 (47.2%)	0.42
	Currently married	84 (57.1%)	113 (52.8%)	
Housing	Other / less secure	22 (15.0%)	22 (10.3%)	< 0.001
	Work-provided	29 (19.7%)	89 (41.6%)	
	Own	96 (65.3%)	103 (48.1%)	
Ethnicity	Kachin	37 (25.2%)	37 (17.3%)	0.19
	Bamar	79 (53.7%)	126 (58.9%)	
	Other incl. Shan and Pa’O	31 (21.1%)	51 (23.8%)	
Age group	15–24	22 (15.0%)	61 (28.5%)	< 0.001
	25–34	49 (33.3%)	104 (48.6%)	
	35–49	76 (51.7%)	49 (22.9%)	
Sexual partner of those who use or inject drugs	No	56 (40.9%)	108 (54.3%)	0.016
	Yes	81 (59.1%)	91 (45.7%)	
	Missing	10 (6.8%)	15 (7.0%)	
Experience of selling sex or drug use	Neither	34 (23.1%)	51 (23.8%)	< 0.001
	Selling sex only	31 (21.1%)	93 (43.5%)	
	Drug use only	49 (33.3%)	32 (15.0%)	
	Both	33 (22.4%)	38 (17.8%)	
Drug group	Never used	65 (44.2%)	144 (67.3%)	< 0.001
	Opioids	35 (23.8%)	19 (8.9%)	
	Stimulants	44 (29.9%)	43 (20.1%)	
	Other	3 (2.0%)	8 (3.7%)	
Survey /clinic site	Site 1 (Kachin State)	37 (%)	31 (%)	0.025
	Site 2 (Kachin State)	74 (%)	105 (%)	
	Site 3 (Kachin State)	20 (%)	41 (%)	
	Site 4 (Shan State)	16 (%)	37 (%)	

Bivariate associations were assessed using Pearson’s chi-squared test

### Multivariable analyses

#### Contraceptive use

Table 2 presents two adjusted models examining factors associated with any contraceptive use in the past 12 months. Model 1 included experience of selling sex or drug use as the main independent variable, while Model 2 instead included drug use group.

In Model 1, compared with participants with neither selling sex nor drug use experience, those with drug use only were significantly less likely to report contraceptive use (aPR 0.68, 95% CI 0.48–0.95). Participants engaged only in selling sex did not differ significantly (aPR 0.89, 95% CI 0.60–1.31).

In Model 2, women who used drugs, both opioid (aPR 0.64, 95% CI 0.43–0.95) and stimulant users (aPR 0.72, 95% CI 0.57–0.92) were significantly less likely to report contraceptive use, while the small group reporting other or rare drug types did not differ (aPR 1.05, 95% CI 0.73–1.51), compared to women who never used drugs.

In Model 1, higher educational attainment was not consistently associated with contraceptive use; only women with university-level education had a higher prevalence compared with those with no formal education (aPR 1.44, 95% CI 1.02–2.03). Similar patterns were observed in Model 2, where university education remained positively associated with contraceptive use (aPR 1.43, 95% CI 1.01–2.01), while primary, secondary, and tertiary education were not.

Housing status showed a modest association with contraceptive use in both models. In Model 1, women living in work-provided housing had a higher prevalence of contraceptive use compared with those in other or less secure housing (aPR 1.43, 95% CI 1.01–2.05); a similar but borderline association was observed in Model 2 (aPR 1.40, 95% CI 0.99–1.96). This association was driven primarily by women engaged in selling sex, either alone or in combination with drug use, who comprised approximately 96% of participants residing in work-provided housing. In this context, work-provided housing likely reflects brothel-based residence or other work-linked settings, where contraceptive access may be facilitated through proximity to outreach services, peer networks, or employer- or program-mediated provision. Estimates among the small number of women not engaged in selling sex who reported work-provided housing were imprecise and should be interpreted with caution.

Marital status was positively associated with contraceptive use in both models. Compared with women who were not currently married, currently married women had a higher prevalence of contraceptive use in Model 1 (aPR 1.36, 95% CI 1.12–1.65) and Model 2 (aPR 1.37, 95% CI 1.13–1.66).

No statistically significant differences in contraceptive use were observed by ethnicity in either model. Compared with Kachin women, contraceptive use did not differ significantly among Bamar women or women from other ethnic groups, including Shan and Pa’O.

Age was strongly associated with contraceptive use in both models. Women aged 35–49 years were significantly less likely to report contraceptive use compared with those aged 15–24 years (Model 1: aPR 0.62, 95% CI 0.47–0.82; Model 2: aPR 0.65, 95% CI 0.49–0.86), while no significant differences were observed between women aged 25–34 years and the youngest age group.

**Table 2:** Factors associated with contraceptive use using selling sex/drug-use status and drug type models, multivariable analyses

Variable	Category	Model 1 PR (95% CI)	Model 2 PR (95% CI)
Experience of selling sex or drug use	Neither	Ref.	–
	Sex work only	0.89 (0.60–1.31)	–
	Drug use only	0.68* (0.48–0.95)	–
	Both	0.69 (0.47–1.02)	–
Sexual partner uses/injects drugs	No	Ref.	Ref.
	Yes	0.96 (0.75–1.23)	0.98 (0.79–1.21)
Education	No formal education	Ref.	Ref.
	Primary school	0.90 (0.62–1.29)	0.87 (0.61–1.24)
	Secondary school	1.13 (0.81–1.58)	1.12 (0.81–1.56)
	Tertiary school	1.10 (0.80–1.52)	1.07 (0.78–1.47)
	University level	1.44* (1.02–2.03)	1.43* (1.01–2.01)
Housing	Other / less secure	Ref.	Ref.
	Work-provided	1.43* (1.01–2.05)	1.40 (0.99–1.96)
	Own	0.99 (0.73–1.34)	0.98 (0.72–1.32)
Ethnicity	Kachin	Ref.	Ref.
	Bamar	0.92 (0.70–1.22)	0.93 (0.71–1.22)
	Other (incl. Shan, Pa’O)	1.14 (0.85–1.53)	1.17 (0.87–1.57)
Age group (years)	15–24	Ref.	Ref.
	25–34	0.99 (0.83–1.18)	1.02 (0.86–1.21)
	≥ 35	0.62*** (0.47–0.82)	0.65** (0.49–0.86)
Employment	No	Ref.	Ref.
	Yes	1.09 (0.84–1.41)	1.07 (0.83–1.36)
Survey / clinic site	Site 1 (Kachin State)	Ref.	Ref.
	Site 2 (Kachin State)	1.10 (0.80–1.53)	1.04 (0.76–1.44)
	Site 3 (Kachin State)	1.45* (1.05–2.01)	1.36 (0.98–1.89)
	Site 4 (Shan State)	1.14 (0.78–1.66)	1.04 (0.72–1.51)
Marital status	Not currently married	Ref.	Ref.
	Currently married	1.36** (1.12–1.65)	1.37** (1.13–1.66)
Drug group	Never used	–	Ref.
	Opioids	–	0.64* (0.43–0.95)
	Stimulants	–	0.72** (0.57–0.92)
	Other / rare	–	1.05 (0.73–1.51)
N		336	336

PR, prevalence ratio; CI, confidence interval. Model 1 includes experience of selling sex or drug use; Model 2 includes drug group instead of experience categories. *p < 0.05; **p < 0.01; ***p < 0.001

### Condom use with different partner types

Table 3 presents adjusted prevalence ratios (aPRs) for condom use across four separate multivariable Poisson regression models corresponding to partner type: main (Model 1), regular (Model 2), casual known (Model 3), and casual unknown partners (Model 4). In all models, the reference category for the main exposure was women with experience of selling sex only.

For main and regular partners (Models 1–2), condom use did not differ significantly by experience of selling sex or drug use. Living in work-provided housing was associated with higher condom use with main partners (aPR 2.29, 95% CI 1.07–4.90), while the association for regular partners was of similar magnitude but not statistically significant (aPR 2.51, 95% CI 0.87–7.19). Condom use with main and regular partners was also more common among participants recruited in Site 4 (Shan State) compared with Site 1 (Kachin State) (aPR 2.29, 95% CI 1.16–4.50 and aPR 2.91, 95% CI 1.02–8.33, respectively). No consistent associations were observed for age, education, employment, ethnicity, marital status, or having a partner who injects drugs.

For casual known partners (Model 3), condom use differed markedly by experience of selling sex or drug use. Compared with women who sold sex only, those with no personal experience of selling sex or drug use (aPR 0.38, 95% CI 0.16–0.88) and those with both selling sex and drug use experience (aPR 0.64, 95% CI 0.50–0.81) were significantly less likely to report condom use. Participants aged 25–34 years had higher condom use compared with those aged 15–24 years (aPR 1.27, 95% CI 1.00–1.60). Condom use was substantially lower among participants recruited from Site 3 (Kachin State) (aPR 0.20, 95% CI 0.06–0.73).

For casual unknown partners (Model 4), condom use was strongly patterned by selling sex and drug use experience. Participants with no experience of selling sex or drug use (aPR 0.18, 95% CI 0.05–0.65) and those reporting drug use only (aPR 0.23, 95% CI 0.08–0.69) were substantially less likely to report condom use compared with women who sold sex only. In contrast, women reporting both selling sex and drug use had slightly lower condom use than the reference group, though the magnitude was modest (aPR 0.87, 95% CI 0.76–0.99). Condom use with casual unknown partners was higher among participants aged 25–34 years (aPR 1.16, 95% CI 1.01–1.33) and lower among those recruited in Site 4 (Shan State) compared with Site 1 (aPR 0.52, 95% CI 0.30–0.88).

Across all condom use models, no statistically significant associations were observed for education level, employment status, ethnicity, marital status, or reporting a sexual partner who injected drugs.

**Table 3:** Factors associated with condom use in the past 3 months by partner type, multivariable analysis

Variable	Category	Model n1 PR (95% CI)	Model n2 PR (95% CI)	Model n3 PR (95% CI)	Model n4 PR (95% CI)
Experience of selling sex or drug use	Sex work only	Ref.	Ref.	Ref.	Ref.
	Neither	1.07 (0.45–2.51)	1.26 (0.43–3.75)	0.38* (0.16–0.88)	0.18** (0.05–0.65)
	Drug use only	1.02 (0.49–2.12)	1.02 (0.40–2.64)	0.38 (0.13–1.07)	0.23** (0.08–0.69)
	Both	0.94 (0.56–1.58)	0.89 (0.46–1.74)	0.64*** (0.50–0.81)	0.87* (0.76–0.99)
Sexual partner injects drugs	No	Ref.	Ref.	Ref.	Ref.
	Yes	0.91 (0.53–1.58)	0.89 (0.44–1.81)	0.90 (0.66–1.21)	0.99 (0.84–1.17)
Education	No formal education	Ref.	Ref.	Ref.	Ref.
	Primary school	1.23 (0.57–2.64)	1.32 (0.49–3.50)	1.08 (0.76–1.54)	1.02 (0.86–1.20)
	Secondary school	1.06 (0.49–2.26)	0.98 (0.36–2.67)	1.07 (0.75–1.53)	1.00 (0.84–1.19)
	Tertiary school	1.53 (0.69–3.36)	1.71 (0.60–4.83)	0.97 (0.68–1.39)	1.09 (0.93–1.28)
	University level	1.61 (0.59–4.39)	2.01 (0.56–7.22)	0.73 (0.38–1.40)	0.76 (0.46–1.25)
Housing	Other / less secure	Ref.	Ref.	Ref.	Ref.
	Work-provided	2.29* (1.07–4.90)	2.51 (0.87–7.19)	0.80 (0.28–2.30)	1.19 (0.80–1.78)
	Own	1.23 (0.60–2.53)	1.14 (0.40–3.26)	0.46 (0.16–1.32)	0.76 (0.49–1.16)
Ethnicity	Kachin	Ref.	Ref.	Ref.	Ref.
	Bamar	1.08 (0.63–1.87)	1.39 (0.59–3.24)	0.79 (0.50–1.27)	0.96 (0.75–1.23)
	Other (incl. Shan, Pa’O)	1.22 (0.67–2.22)	1.66 (0.68–4.07)	0.94 (0.57–1.55)	1.13 (0.86–1.48)
Age group (years)	15–24	Ref.	Ref.	Ref.	Ref.
	25–34	0.98 (0.67–1.43)	1.01 (0.60–1.70)	1.27* (1.00–1.60)	1.16* (1.01–1.33)
	≥ 35	0.79 (0.46–1.38)	0.91 (0.43–1.93)	1.16 (0.70–1.93)	0.93 (0.70–1.22)
Employment	No	Ref.	Ref.	Ref.	Ref.
	Yes	0.77 (0.49–1.19)	0.81 (0.45–1.47)	0.85 (0.66–1.10)	0.87 (0.75–1.01)
Survey/clinic site	Site 1 (Kachin State)	Ref.	Ref.	Ref.	Ref.
	Site 2 (Kachin State)	1.38 (0.74–2.54)	1.99 (0.77–5.19)	1.28 (0.48–3.43)	0.82 (0.53–1.26)
	Site 3 (Kachin State)	0.59 (0.26–1.33)	0.57 (0.15–2.13)	0.20* (0.06–0.73)	0.73 (0.46–1.14)
	Site 4 (Shan State)	2.29* (1.16–4.50)	2.91* (1.02–8.33)	0.79 (0.29–2.15)	0.52* (0.30–0.88)
Marital status	Not currently married	Ref.	Ref.	Ref.	Ref.
	Currently married	1.03 (0.68–1.56)	1.02 (0.60–1.74)	1.06 (0.87–1.30)	0.93 (0.81–1.08)
N		294	294	154	191

Each column corresponds to a separate multivariable Poisson regression model for a distinct outcome. Condom use was assessed through separate questionnaire items for each partner type (main, regular, casual known, casual unknown); these were therefore analyzed as different outcomes. Sample size varies across condom use models because only participants who reported having the corresponding partner type were asked about condom use and contributed to that analysis. As the analyses do not involve repeated testing of the same outcome, no adjustment for multiple testing was applied

*p < 0.05, **p < 0.01, ***p < 0.001

## Discussion

This study examined factors associated with contraceptive and condom use among women who sell sex, use drugs, or are partners of men who use or inject drugs in Myanmar. Across outcomes, contraceptive and condom use were shaped primarily by risk group membership and structural conditions rather than by sociodemographic characteristics alone. These findings are consistent with the “risk environment” framework, which emphasizes that health behaviors and outcomes are shaped by interactions between individual agency and structural conditions such as criminalization, policing, housing instability, stigma, and differential access to services [8, 9]. This perspective is particularly relevant to women who sell sex or use drugs, whose access to prevention and reproductive health services may depend not only on individual behavior but also on their position within social, economic, and service environments.

### Contraceptive use

Women who used drugs, particularly those reporting drug use only, had a lower prevalence of contraceptive use compared with women with no personal experience of selling sex or drug use. In contrast, women engaged only in selling sex did not differ significantly from the reference group, which may indicate that participation in sex work–focused outreach and service networks can help reduce some barriers to contraceptive access. These findings may partly reflect differences in access to and engagement with outreach and SRHR services: women who sell sex in Myanmar may have greater contact with peer outreach and targeted sexual and reproductive health services through community-based programs while women who use drugs but do not sell sex may have fewer entry points to family planning services. Similar concerns have been identified internationally, where reproductive health services are often insufficiently integrated into harm reduction programs, while peer-led and sex worker-led interventions have provided important platforms for condom distribution and broader SRHR support [12].

Beyond service access, prior research suggests that limited contraceptive knowledge, partner influence on reproductive decision-making, and co-occurring health concerns may further constrain contraceptive uptake among women who use drugs, although these mechanisms were not directly assessed here. This aligns with evidence that women who use drugs face persistent barriers to SRHR services due to stigma, criminalization, and limited integration of reproductive health within harm reduction programs [1, 14–16, 31–34]. Taken together, these findings suggest that lower contraceptive use among women who use drugs may reflect multiple intersecting barriers rather than drug use itself as an isolated determinant.

Women without personal experience of selling sex or drug use, many of whom were sexual partners of men who use or inject drugs, also did not demonstrate higher contraceptive use. Although these women may not be directly engaged with high-risk environments, they may still experience indirect exposure to reproductive and infection-related risks through their partners. Evidence from other settings suggests that sexual partners of people who use drugs often fall outside the reach of both harm reduction and sex work–focused programs, despite ongoing vulnerability [6, 17, 35]. Their position therefore illustrates a potential gap in conventional keypopulation programming: women may experience risks associated with drug-use networks without themselves being classified as people who use drugs or as sex workers. In Myanmar, organizations such as AHRN and Best Shelter do provide some services to partners of people who use drugs; however, these findings suggest that additional, more tailored outreach strategies are needed to ensure adequate contraceptive coverage for this group.

Housing stability emerged as a consistent structural factor. Women residing in work-provided housing had a higher prevalence of contraceptive use, although estimates were borderline in one model. This association was driven primarily by women engaged in selling sex, either alone or in combination with drug use, who comprised approximately 96% of residents in work-provided housing. In this context, “work-provided” housing likely reflects brothel-based or work-linked residence. Rather than indicating that work-provided housing itself improves contraceptive use, this finding may reflect the social and service environment associated with such settings. Such environments may function as informal structural intervention settings where outreach services, peer networks, and condom/contraceptive distribution are more readily available, consistent with findings from sex worker community-led interventions in Asia and globally [7, 10, 21, 36–38]. At the same time, the cross-sectional design prevents us from determining whether housing facilitated service access or whether other characteristics of women living in these settings explain the association.

Older women (aged 35–49 years) were significantly less likely to use contraception, consistent with evidence from Myanmar and other low- and middle-income countries showing lower coverage of demand for family planning satisfied with modern methods among older age groups [39]. This finding suggests that reproductive health programming for marginalized women should not assume that younger age alone defines unmet need and should consider age-specific barriers to contraceptive access and use.

Drug group analyses further highlighted disparities. Women using opioids or stimulants were significantly less likely to report contraceptive use compared with women who had never used drugs. The consistency of this pattern across drug groups suggests that barriers may extend beyond the specific substance used and may instead reflect broader social and service environments associated with drug use. This pattern aligns with AHRN’s operational experience, which indicates that women who use stimulants, in particular, often experience high mobility, unstable living conditions, and limited engagement with routine health services. Many are economically active during clinic hours, reducing opportunities to access facility-based family planning services. This is consistent with global evidence showing that stimulant-using women are particularly vulnerable to disrupted health service engagement due to mobility, economic insecurity, and episodic care access patterns. These findings reinforce the need for flexible, community-based SRHR delivery models tailored to the lived realities of women who use drugs [7, 10, 17, 31, 32, 34, 40]. Flexible, community-based SRHR delivery may therefore be particularly important for women whose circumstances make routine facility-based care difficult to access.

### Condom use by partner type

For main and regular partners, condom use did not differ significantly by selling sex or drug use experience, indicating that the relationship between risk-group membership and condom use may depend on the type of sexual partnership. Condom negotiation in more stable partnerships may be influenced by relationship dynamics, trust, perceived HIV risk, or reproductive intentions, rather than by program exposure alone. This contrasts with the clearer differences observed for casual partnerships and underscores the importance of considering partner type when evaluating HIV-prevention behaviors.

In contrast, condom use with casual partners was strongly patterned by selling sex and drug use experience. Women with no experience of selling sex or drug use and those reporting drug use only were substantially less likely to report condom use compared with women who sold sex. Women reporting both selling sex and drug use also had lower condom use with casual partners, although the magnitude was smaller. One possible explanation is differential exposure to condom promotion, peer outreach, and HIV-prevention services, as women engaged in selling sex may have more regular contact with targeted prevention programs. Previous research has similarly highlighted the importance of peer-led and community-based approaches in reaching women who sell sex and people who use drugs, while also identifying persistent gaps among populations who fall outside established program categories [11, 12, 18–20]. Our findings extend this literature by showing that these differences may also emerge according to partner type, with women outside sex work–focused outreach potentially less likely to use condoms with casual partners.

The particularly low condom use observed among women with no personal experience of selling sex or drug use is important because these women may not be readily identified through conventional keypopulation programming despite potential exposure to HIV and STI risks through their sexual networks. This highlights the need for prevention strategies that complement population-based targeting with approaches capable of reaching women whose risk is less visible through conventional program categories.

Across all condom use models, no consistent associations were observed for education, employment, ethnicity, marital status, or having a partner who injected drugs. This reinforces the importance of situational and structural factors such as service access, housing context, and outreach exposure over individual sociodemographic characteristics in shaping protective behaviors [8, 9, 41, 42]. However, these findings should not be interpreted as evidence that sociodemographic characteristics are unimportant; rather, their effects may be mediated through the structural and service environments in which women make reproductive and HIV-prevention decisions.

### Broader structural and policy context and future research

These findings must be interpreted within Myanmar’s current political and economic context, including deteriorating health service access following the 2021 coup and ongoing humanitarian constraints [43–47]. Similar disruptions have been associated with reduced continuity of harm reduction and SRHR services in other conflict-affected settings, where NGO-led systems become primary service providers but remain unevenly distributed [13, 22, 48]. These broader constraints are particularly relevant for marginalized women, who may already face barriers to conventional health services. The implications are particularly concerning given Myanmar’s HIV epidemiology [49, 50], reinforcing the need for sustained and integrated prevention approaches.

While the risk environment framework highlights how structural conditions shape health risks and access to care, a syndemic perspective provides a complementary way of understanding how multiple health and social problems can cluster and interact. A syndemic refers to the co-occurrence of two or more health conditions or social vulnerabilities whose interaction contributes to worse health outcomes than would be expected from their independent effects [42, 51]. Among women who use drugs or sell sex, substance use, violence, stigma, HIV risk, and socioeconomic insecurity may occur together and reinforce barriers to health and care [51–53]. In this study, the lower contraceptive use observed among women who use drugs and lower condom use with casual partners among some groups may be consistent with such intersecting vulnerabilities, but our data do not allow us to establish these pathways. Because violence, stigma, and several dimensions of social vulnerability were not directly measured, these mechanisms should be regarded as hypotheses rather than demonstrated explanations. Future research could therefore use longitudinal and mixed-methods approaches to examine whether combinations of social, health, and structural vulnerabilities produce mutually reinforcing effects on reproductive and HIV-prevention outcomes.

Differences in exposure to outreach and SRHR services represent another potentially important explanation for the observed patterns. Participants were recruited through AHRN-BS, which provides community-based harm reduction and women-focused services, including SRHR interventions, and information on previous contact with outreach workers was collected. However, outreach exposure was not included in the present analyses, and we therefore cannot determine whether differences in service exposure contributed to the associations observed. Future studies should examine the frequency, type, and timing of outreach contact, including contraceptive and condom provision and referrals to SRHR services, and assess whether these factors mediate or modify associations between risk-group membership and reproductive health outcomes.

Quantitative studies alone cannot fully capture how women navigate reproductive health decisions within these complex social and service environments. Ethnographic and qualitative research could provide particularly valuable insight into participants’ everyday experiences, including how drug use, selling sex, relationships, housing, mobility, stigma, violence, and interactions with health and harm reduction services shape contraceptive decision-making and condom use. Such approaches could also identify barriers and facilitators that are not readily captured through standardized questionnaires and inform the design of more responsive interventions.

### Implications for harm reduction

At the same time, ongoing cuts to overseas development assistance threaten the sustainability of harm reduction, HIV prevention, and SRHR programs [54]. Given these constraints, large-scale expansion of integrated services is unlikely in the near future. Moreover, effective harm reduction should not be understood simply as increasing the volume of resources or services available. The quality, accessibility, acceptability, continuity, and gender responsiveness of services are equally important, particularly for women whose needs may not align with existing program structures. Effective responses should therefore be adapted to the specific social and structural circumstances of women who use drugs and sell sex. This includes strengthening referral pathways between specialized and general SRHR services, ensuring continuity of community-based care, and involving affected communities in the design, implementation, and evaluation of interventions. Such approaches may be particularly important in settings where resources are constrained, and services must be designed to reach populations that remain underserved by conventional harm reduction and SRHR systems.

### Strengths and limitations

Key strengths of this study include its focus on an under-researched and marginalized population and the inclusion of multiple types of sexual partnerships in the analysis. Several limitations should be considered when interpreting the findings. First, the cross-sectional design precludes causal inference, and observed associations should not be interpreted as causal effects. Second, contraceptive and condom use were self-reported and may be affected by social desirability bias, particularly among women whose sexual and drug-use behaviors may be stigmatized or criminalized. Third, some subgroups, particularly those included in partner-specific condom analyses, were small, which may have limited statistical power and the precision of estimates. Fourth, participants were recruited using non-random sampling through community-based outreach, limiting the representativeness of the sample and the generalizability of findings to women in the broader population. Finally, the study covered four sites in Northern and Eastern Myanmar and therefore may not capture the experiences of women in other regions or settings.

## Conclusion

These findings highlight the importance of strengthening access to integrated reproductive health and HIV-prevention services across harm reduction, sex work outreach, and broader sexual and reproductive health systems. The observed differences in contraceptive and condom use may reflect the interaction of risk-group membership with structural conditions and differential access to services, although these mechanisms require further investigation. Future research should directly examine exposure to outreach and SRHR services, as well as the intersecting social and structural conditions that shape reproductive health behaviors. Strengthening services should therefore focus not only on expanding provision, but also on ensuring that interventions are accessible, gender-responsive, community-informed, and able to reach women who remain underserved by existing programs.

## Data Availability

Due to the sensitive nature of the data and confidentiality concerns for participants in conflict-affected areas, the datasets generated and analyzed during this study are not publicly available. Access to de-identified data may be considered on a case-by-case basis upon reasonable request to Asian Harm Reduction Network–Best Shelter (contact@ahrnmyanmar.org), subject to ethical and data protection approvals.

## Abbreviations

AHRN-BS: Asian Harm Reduction Network–Best Shelter
aPR: Adjusted prevalence ratio
CI: Confidence Interval
HIV: Human immunodeficiency virus
SRHR: Sexual and Reproductive Health and Rights
STI: Sexually transmitted infection
